# Risk Factors of CVD in Different Ethnic Groups in Kyrgyzstan

**DOI:** 10.1002/puh2.70025

**Published:** 2025-02-07

**Authors:** Hossain Syed Azfar, Muiz Ibrahim, Kenesh Dzhusupov, Hans Orru, Inga Villa, Kati Orru

**Affiliations:** ^1^ Department of Introduction to Internal Medicine and Family Medicine International Higher School of Medicine Bishkek Kyrgyzstan; ^2^ Department of Public Health International Higher School of Medicine Bishkek Kyrgyzstan; ^3^ Department of Public Health Osh State University Osh Kyrgyzstan; ^4^ Institute of Family Medicine and Public Health University of Tartu Tartu Estonia; ^5^ Institute of Social Studies University of Tartu Tartu Estonia

**Keywords:** alcohol, body mass index (BMI), cardiovascular disease (CVD), ethnic group, Kyrgyzstan, lifestyle

## Abstract

**Introduction:**

Cardiovascular disease (CVD) is the leading cause of mortality in Kyrgyzstan. The study aimed to explore the CVD prevalence depending on various risk factors across diverse ethnic groups in Kyrgyzstan.

**Materials and Methods:**

A cross‐sectional study was carried out among six ethnic groups in Kyrgyzstan, aged 18 years and above. The sample was stratified for age, education, family status, and income. We used a questionnaire with 47 questions to explore the health status, behavior and lifestyle determinants, and prevalence of CVD. We used the chi‐square test to investigate differences between groups, and ANCOVA to determine differences between mean scores on analyzed variables. Logistic regression was used to analyze the relationship between independent and dependent variables. Confounding variables were only included if they correlated with both the independent and dependent variables. Interaction analyses were conducted with logistic regression to investigate if there were any differences between the nationalities in the relationships between the independent and dependent variables.

**Results:**

Our study confirmed that the CVD prevalence across diverse ethnic groups can be significantly different: In Kyrgyzstan, the most disadvantaged groups in this context were East European and the least—Western Asian. There was no difference between studied groups found in the ethnicity‐stratified prevalence of such CVD risk factors as low fruit and vegetable consumption, alcohol intake, smoking, and body mass index (BMI). There was no statistically significant association between educational attainment and CVD risk markers within the studied ethnic groups. The analysis of ethnicity‐stratified prevalence of CVD risk markers resulted in a significant difference in physical activity across ethnicity groups.

**Conclusion:**

The study results provided an understanding of the ethnicity‐stratified prevalence of CVD risk markers in the population in Kyrgyzstan. They could serve as instrumental in tailoring targeted public health interventions to address the burden of CVDs in specific subpopulations.

## Graphical abstract

This study examines CVD risk factors across ethnic groups in Kyrgyzstan, revealing significant disparities in prevalence rates (20.6%–42.7%). Age emerged as a critical factor, with BMI and alcohol consumption identified as significant risk factors across all ethnic groups.

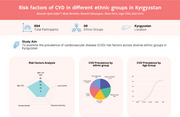

## 1 Introduction

Cardiovascular diseases (CVDs) are the leading cause of death, responsible for 17.9 million or 32% of global deaths in 2019 [1]. Among 51 European and Central Asian countries (WHO European Region), Kyrgyzstan has the 3rd highest CVD mortality rate among women and 5th among men [2]. In Kyrgyzstan, more than 19,000 people die each year due to CVD. The mortality rate from CVD accounted for 50.8% of all deaths in the country in 2013 [3]. In the WHO European region, Kyrgyzstan had the highest coronary heart disease (CHD) mortality rate in 2013 and was the only country with an increased CHD mortality rate [4]. Nevertheless, from 2009 to 2019, there was almost a 5% decrease in the total mortality rate due to ischemic heart disease and more than 13% due to stroke [5]. However, the absence of specialists and portable tools in rural Kyrgyzstan might lead to underreporting of CVD prevalence in the country [6].

Numerous studies have shown the relationships between ethnicity/race and various modifiable CVD risk factors [7, 8, 9]. There are only a few studies on differences in the CVD prevalence between Kyrgyz and Russian patients in Kyrgyzstan with little information on risk factors [10, 11, 12]. Considering the value of knowledge of social determinants of health, particularly ethnicity and behavioral risk factors for further research, policy, and the development of public health and social measures in the prevention of CVDs among the multinational population of Kyrgyzstan, there is a need to explore various determinants of CVDs in different ethnic groups of Kyrgyzstan as a country with one of the highest CVD prevalence worldwide.

**The present study is aimed at** the examination of the main risk factors of CVD, such as dietary habits, physical activity, alcohol use, and smoking among different ethnic groups in Kyrgyzstan.

## 2 Participants and Methods

### 2.1 The Study Site

Kyrgyzstan is located in Central Asia with a population of 6.75 million [13], making it an interesting case study area for analysis of CVD due to its past societal developments and the current mixture of ethnic and cultural groups. Communist rule ended in 1991, and independent Kyrgyzstan has improved its regulatory system and progressed with market reforms. However, the Gini coefficient for Kyrgyzstan was 29 in 2019, indicating relatively well‐distributed income or consumption expenditure among individuals or households (World Bank 2020). Ethnic groups in the country include 74.1% Kyrgyzs, 14.8% Uzbeks, 5.0% Russians, 1.1% Dungans, 0.9% Uyghurs, 0.9% Tajiks, 0.7% Turks, 0.6% Kazakhs, 0.4% Tatars, 0.3% Azeris, 0.3% Koreans, 0.1% Ukrainians, 0.1% Germans, and 0.7% others [13].

### 2.2 Study Design and Data Collection

A cross‐sectional study was conducted among individuals aged 18–60 years and older who participated in a study called “The health status of ethnic minorities in Kyrgyzstan” at polyclinics (Centers of Family Medicine) and healthcare centers in a study called “The health status of ethnic minorities in Kyrgyzstan.” The sample of 694 participants was selected to capture a representative distribution across six major ethnic groups in Kyrgyzstan, ensuring meaningful analysis of CVD prevalence across these subpopulations. This stratified sampling approach provided adequate subgroup sizes for each ethnic group, given the study's logistical constraints and available resources.

**FIGURE 1:**
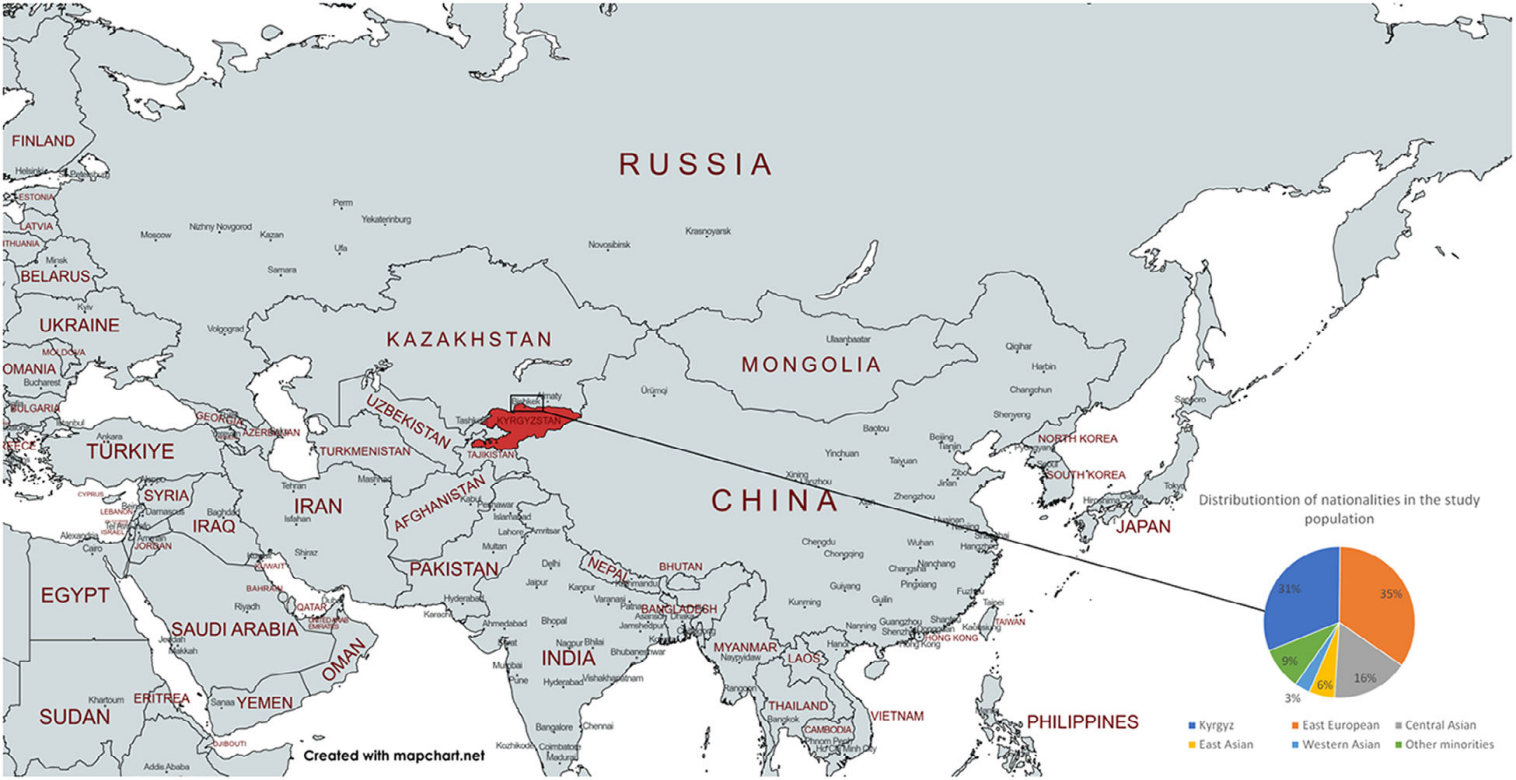
Distribution of ethnic groups in the study sample.

We used a questionnaire with 47 questions to explore the health status, behavior, lifestyle determinants, and prevalence of CVD. Respondents could ask for assistance or explanations from the study leader. Kyrgyz people were taken as a controlled group compared with other ethnic groups. Due to their religious background and geographical origins, we grouped the ethnic groups as follows: (1) East Europeans: Russian, Byelorussian, and Ukrainian; (2) Other Central Asians: Uzbek, Kazakh, Tatar, and Uyghur; (3) East Asians: Korean and Dungan; (4) Western Asians: Georgian, Armenian, Turk, and Azerbaijani; and (5) Other minorities: Dungan, Uyghur (Figure 1). Collected information included age, gender, education level, nationality, family status, income, dietary habits (fruits, vegetables, alcohol), physical activity, smoking, and body mass index (BMI). The sample was stratified for age, education, family status, and income. Prior to data collection, a power analysis was conducted to determine the minimum sample size required for detecting significant differences in CVD prevalence across ethnic groups. On the basis of the effect sizes derived from similar studies in Central Asia, we estimated that a sample size of 694 would provide an 80% power to detect moderate differences in CVD risk factors, such as BMI, physical activity, and dietary habits, among the ethnic groups.

### 2.3 Questions and Questionnaire Instruments

The frequency of food and alcohol consumption was given per day, per week, and per month, depending on the food item and drinking. Portion sizes were units such as glasses and cups, and the portion sizes of the different food items were converted on the basis of the standard portions. We also included questions about weight, height, physical activity, and smoking habits. The specific questions are described as follows:

Dietary habits were measured by two questions: (A) “How often do you eat fruits or berries?” (B) “How often do you eat vegetables and salads (except vegetable juices and potatoes)?”

Alcohol consumption was measured with: (A) “How often do you drink alcohol?” (B) “How many glasses of alcohol do you drink on a typical occasion? One ‘glass’ means 50 cl light beer, 33 cl strong beer, 1 glass of red or white wine, 1 small glass of strong wine, or 4 cl spirit (vodka, whiskey).”

We regrouped the variable: Alcohol consumption: Males who consume at least 2–4 times a month at least 5–6 glasses of alcohol as a risk group; all other less frequent male drinkers as referents. Females who consume at least 2–4 times a month at least 3–4 glasses of alcohol as the risk group; all other less frequent female drinkers as referents.

The question of smoking was: “Do you smoke?” We regrouped the responses: from 1 to 3 as (2)—risk group; and 4 as (1)—referents.

Questions on physical activity included: (A) “During the last 7 days, how many days did you do vigorous physical activities like heavy lifting, digging, aerobics, or fast bicycling? Days per week.” (B) “How much time did you usually spend doing vigorous physical activities on one of those days? Hours per day, minutes per day, don't know/Not sure.” Responses were converted to minutes per week. (C) “During the last 7 days, how many days did you do moderate physical activities like carrying light loads, bicycling at a regular pace, or doubles tennis? Do not include walking; days per week.” (D) “How much time did you usually spend doing moderate physical activities on one of those days? Hours per day, minutes per day? Do not know/Not sure.” Responses were converted to minutes per week. (E) “During the last 7 days, on how many days did you walk for at least 10 min at a time? Days per week.” (F) “During the last 7 days, how much time did you spend sitting on a weekday? Hours per day, minutes per day.” Responses were converted to minutes per week.

To calculate total physical activity, the minutes of vigorous physical activity were doubled to equal the minutes of moderate physical activity. Thereafter, the minutes of vigorous and moderate physical activity, as well as walking, were summarized. Individuals with 150 or more minutes of moderate physical activity per week were grouped as (1)—referents; and individuals with less physical activity as (2)—risk group.

Individuals were regrouped according to their BMI as follows: BMI < 25 as (1)—referents; BMI ≥ 25 kg/m^2^ as (2)—risk group.

### 2.4 Statistical Analysis

We used the chi‐square test to investigate differences between groups and ANCOVA to determine whether there were significant differences between mean scores on analyzed variables. Logistic regression was used to analyze the relationship between independent variables (nationality, education, family status, income, dietary habits, physical activity, alcohol use, and smoking) and the dependent variable (CVD). Confounding variables were included if they correlated with both the independent and dependent variables. Interaction analyses were conducted with logistic regression to investigate if there were differences between the nationalities in the relationships between the independent and dependent variables. A *p*‐value of <0.05 was considered statistically significant for all statistical tests.

Post hoc pairwise tests were performed to identify specific significant differences between groups for comparisons among education, age, income, or ethnic groups. Bonferroni correction was applied to adjust for multiple comparisons, ensuring robust significance determination at *p* < 0.05.

## 3 Results

The prevalence of CVDs in different sociodemographic groups is presented in Table 1.

**TABLE 1:** Prevalence of cardiovascular disease in different sociodemographic groups (%), difference between groups.

Factor risks	CVD, % (*n*)	No CVD, % (*n*)	Chi‐square	*p* = value
**Gender**			28.26	0.000^*^
Male	22.8 (69)	77.2 (233)		
Female	42.1 (165)	57.9 (227)		
**Age**			373.37	0.000^*^
18–29	1.7 (3)	98.3 (170)		
30–39	4.7 (8)	95.3 (161)		
40–49	30.0 (36)	70.0 (84)		
50–59	76.7 (92)	23.3 (28)		
≥60	84.8 (95)	15.2 (17)		
**Education**			8.372	0.039^*^
Primary school	0.9 (2)	0 (0)		
Secondary school	3.7 (8)	8.6 (11)		
High school (vocational)	49.1 (106)	57.0 (73)		
University degree	46.3 (100)	34.4 (44)		
**Family status**			0.37	0.847
Single	33.3 (104)	66.7 (208)		
Married or cohabiting	34.0 (130)	66.0 (252)		
**Nationality**			17.29	0.004^*^
Kyrgyz	30.9 (67)	69.1 (150)		
East European^a^	42.7 (102)	57.3 (137)		
Central Asian	26.3 (30)	73.7 (84)		
East Asian	34.2 (13)	65.8 (25)		
Western Asian	39.1 (9)	60.9 (14)		
Other minorities^a^	20.6 (13)	79.4 (50)		
**Income (KGS, Kyrgyz soms)**			27.8	0.000^*^
≤8000	57.6 (83)	42.4 (61)		
8001–16,000	38.7 (53)	61.3 (84)		
16,100–30,000	31.8 (35)	68.2 (75)		
≥30,001	26.3 (21)	73.8 (59)		

*Note:* Superscript letter (a) indicates statistically significant differences between specific subgroups (*p* < 0.05).

Abbreviation: CVD, cardiovascular disease.

* indicates a statistically significant difference between groups (*p* < 0.05).

There is significant evidence of different CVD prevalence in gender groups (chi‐square = 28.26, *p* < 0.000), with higher prevalence among males. There is also a significant difference in CVD prevalence among different age groups (Chi‐square = 373.37, *p* < 0.000). Among individuals with CVD, 13% are 18–29 years old, 34% are 30–39 years old, 15.4% are 40–49 years old, 39.3% are 50–59 years old, and 40.6% are 60 years old and above. Education level showed a marginally significant association with CVD prevalence (*p* = 0.039), with higher CVD rates observed among participants with lower educational attainment. There is no significant difference in CVD prevalence among single and cohabiting individuals (chi‐square = 0.37, *p* = 0.847). The pairwise comparison within age, education, and income groups did not show any significant disparity after the Bonferroni correction.

There is a significant difference in CVD prevalence among different ethnicity groups (chi‐square = 17.29, *p* < 0.004). Among individuals with CVD, 28.6% are Kyrgyz; 43.6%—East European; 12.8%—of Central Asian origin; 5.6%—East Asians; 3.8%—Western Asians; and 5.6% are other minorities. Although there was a difference with a chi‐square result suggesting some disparity between Kyrgyzs and East Europeans, it was insignificant after the Bonferroni correction. After applying the Bonferroni correction, the pairwise comparisons among ethnic groups showed statistically significant differences in CVD prevalence only between East Asians and other minorities.

There is a notable difference in CVD prevalence between income groups (chi‐square = 27.8, *p* < 0.000). CVD appears among 43.2% of individuals who earn ≤8000 KGS; 27.6% among individuals who earn between 8001 and 16,000 KGS; 18.2% among individuals who earn 16,001–30,000 KGS; and 10.9% among individuals who earn ≥30,001 KGS.

The prevalence of specific cardiovascular conditions, such as hypertension (HT) and CHD, was examined within the sample. HT was observed in 212 cases, which made up 89.1% of participants with CVDs. One hundred seventy‐three respondents (72.8% of the study population with CVDs) reported CHD. The additional analysis of specific CVD conditions, such as HT and CHD, highlights the high prevalence of HT across all ethnic groups, with particularly elevated rates in the Eastern Asian group. They made up 41.9% of all respondents with CVDs (89 out of 212).

The prevalence of CVD in different lifestyle groups is given in Table 2.

**TABLE 2:** Prevalence of cardiovascular disease in different **lifestyle** groups (%), the difference between groups.

Factor risks	CVD, % (*n*)	No CVD, % (*n*)	Chi‐square	*p*‐value
**Fruits**			0.04	0.828
Referents	35.9 (84)	36.7 (169)		
Fruit intake deficit group	64.1 (150)	63.3 (291)		
**Vegetables**			0.24	0.621
Referents	34.6 (81)	36.5 (168)		
Vegetable intake deficit group	65.4 (153)	63.5 (292)		
**Physical activity**			2.72	0.099
Referents	56.8 (133)	50.2 (231)		
Physically inactive group	43.2 (101)	49.88 (229)		
**BMI**			62.91	0.000
<25kg/m^2^	30.3 (71)	62.2 (286)		
≥25 kg/m^2^	69.7 (163)	37.9 (174)		
**Alcohol**			3.87	0.049
Referents	94.4 (221)	97.4 (448)		
Risk group	5.6 (13)	2.6 (12)		
**Smoking**			0.02	0.8777
Referents	166 (69.7)	314 (68.9)		
Risk group	72 (30.3)	142 (31.1)		

Abbreviations: BMI, body mass index; CVD, cardiovascular disease.

On the basis of the results of the chi‐square test, there is no significant difference in fruit and vegetable consumption, physical activity, and smoking rate among people with CVD and without CVD in the population studied (Table 2). However, there was a statistically significant difference in BMI index (chi‐square = 62.91, *p* = 0.000) and alcohol consumption (chi‐square = 3.87, *p* = 0.049) between the two studied groups.

Ethnicity‐stratified prevalence of CVD risk markers, the share of individuals in the risk group (%), and differences between groups are presented in Tables 3 and 4.

**TABLE 3:** Ethnicity‐stratified prevalence of cardiovascular disease (CVD) risk factors, the share of individuals in a risk group (%), and the difference between groups.

	Kyrgyz	East European	Central Asian	East Asian	Western Asia	Other minorities	Chi‐square (*p*value)
**Total *N* = 694**	217	239	114	38	23	63	
Fruit intake deficit group	59.9	65.3	64.9	55.3	69.6	69.8	4.22 (0.225)
Vegetable intake deficit group	61.8	66.1	67.5	55.3	65.2	63.5	2.84 (0.938)
Smoking	21.0	37.9	26.3	31.6	17.4	41.0	21.19 (0.055)
Alcohol consumption	3.7	3.8	3.5	2.6	0.0	4.8	1.23 (0.93)
Alcohol less strict	10.5	15.0	9.9	7.9	4.3	14.5	5.13 (0.93)
Physical activity	42.4	47.3	50.9	55.3	39.1	58.7	7.54 (0.029)
BMI	42.9	58.6	46.5	34.2	52.2	41.3	17.22 (0.98)

Abbreviation: BMI, body mass index.

**TABLE 4:** Ethnicity‐stratified prevalence of cardiovascular disease (CVD) risk markers and group differences.

	Kyrgyz	East European	Central Asia	East Asia	Western Asia	Other minorities
**Total *N* = 694**	217	239	114	38	23	63
**Gender**						
Male	16 (23.9)	26 (25.5)	10 (33.3)	8 (61.5)	3 (33.3)	6 (46.2)
Female	51 (76.1)	76 (74.5)	20 (66.7)	5 (38.5)	6 (66.7)	7 (53.8)
Chi‐square (*p*‐value)	5.29 (0.021)	16.82 (0.000)	3.22 (0.073)	0.32 (0.575)	2.10 (0.147)	3.10 (0.078)
Age groups						
18–29	1 (1.5)	0 (0.0)	1 (3.3)	1 (7.7)	0 (0.0)	0 (0.0)
30–39	2 (3.0)	5 (4.9)	0 (0.0)	1 (7.7)	0 (0.0)	0 (0.0)
40–49	14 (20.9)	9 (8.8)	9 (30.0)	0 (0.0)	1 (11.1)	3 (23.1)
50–59	36 (53.7)	34 (33.3)	12 (40.0)	2 (15.4)	4 (44.4)	4 (30.8)
≥60	14 (20.9)	54 (52.9)	8 (100.0)	9 (69.2)	4 (44.4)	6 (46.2)
Chi‐square (*p* value)	111.01 (0.000)	126.66 (0.000)	55.08 (0.000)	17.01 (0.002)	19.50 (0.001)	52.53 (0.000)
Education						
Edu: primary school	2 (3.0)	0 (0.0)	0 (0.0)	0 (0.0)	0 (0.0)	0 (0.0)
Edu: secondary school	2 (3.0)	3 (3.0)	3 (10.0)	0 (0.0)	6 (33.3)	1 (7.7)
Edu: high school (vocational)	27 (40.3)	53 (52.0)	17 (56.7)	9 (69.2)	3 (16.7)	8 (61.5)
Edu: University degree	36 (53.7)	46 (45.0)	10 (33.3)	4 (30.8)	9 (50.0)	4 (30.8)
Chi‐square (*p* value)	3.55 (0.315)	5.30 (0.151)	1.03 (0.795)	0.31 (0.576)	1.59 (0.452)	0.37 (0.832)

## 4 Discussion

The provided data present an analysis of CVD prevalence across different demographic groups in Kyrgyzstan, including gender, age, education level, ethnicity, and income.

### 4.1 Gender and CVD Prevalence

CVD risk profiles of men and women in low‐ and middle‐income countries vary depending on age, education level, and other factors [14, 15, 16, 17]. High blood pressure is a common condition in Kyrgyzstan among both men and women, with a female disadvantage [18]. Our study confirmed a significant association between gender and CVD prevalence, with a significantly higher prevalence in women (chi‐square = 28.26, *p* < 0.000). These results are comparable to earlier research findings from Kyrgyzstan. For instance, Polupanov et al. found a higher prevalence of cardiovascular risk factors among women (68.2%), which is very close to our findings [12]. This suggests a significant variation in the prevalence of CVD between genders in Kyrgyzstan. In the USA, European countries, and South Asia, the prevalence in women is also higher than in men, but with variation [19, 20, 21].

### 4.2 Age and CVD Prevalence

There has been a well‐established association between age and CVD risk factors prevalence [22, 23, 24, 25, 26]. Our study also reveals a significantly higher prevalence of CVD in older age groups.

### 4.3 Educational Attainment and CVD Prevalence

Several studies have shown a negative correlation of CVD with educational attainment [22, 27, 28, 29]. However, a number of studies have not found any association between CVD prevalence, its risk factors, and education level [30]. In our study, we found a statistically significant difference in CVD prevalence based on education level (chi‐square = 8.372, *p* = 0.039). However, if we studied those aged 40–95, we saw among individuals with high education a noticeably higher prevalence. This can be explained by the socioeconomic transition period in Kyrgyzstan, where the wages of knowledge workers are significantly lower than those in other sectors, leading to higher occupational mental stress. These findings indicate the need for further study; nevertheless, educational attainment is not a direct contributor to the CVD prevalence in the studied population.

### 4.4 Income and CVD Prevalence

Our study revealed a notable difference in CVD prevalence between income groups, with CVD more common among individuals with lower incomes. This aligns with earlier studies showing higher CVD prevalence among adults with lower socioeconomic status [16, 31, 32, 33, 34].

### 4.5 Lifestyle Factors and CVD Prevalence

There is strong evidence of the role of lifestyle factors such as diet [3, 8, 28, 35, 36, 37], physical activity [3, 34, 38, 39, 40], alcohol consumption [8, 34, 41], smoking [3, 8, 21, 42, 43, 44], and overweight [21, 45] on CVD outcomes. Our study found no significant association between fruit and vegetable consumption, physical activity rate, and CVD prevalence in the Kyrgyz population (Table 2). However, BMI index and alcohol consumption showed a statistically significant difference between people with and without CVD. This suggests that higher BMI plays a significant role in developing or progressing CVDs in the Kyrgyz population. Higher BMI is a well‐known risk factor for various cardiovascular conditions, including HT, dyslipidemia, and type 2 diabetes [46]. Therefore, individuals with higher BMI might be at greater risk of developing CVD compared to those with a lower BMI in this population.

Additionally, heavy and moderate alcohol intake has been linked to various cardiovascular risks, including HT, cardiomyopathy, and arrhythmias. Thus, individuals with higher alcohol intake may be more likely to develop CVD compared to those with lower alcohol consumption.

Despite the established role of smoking as a significant risk factor for CVD in global populations [42], our study did not find sufficient evidence to conclude a statistically significant difference in smoking prevalence (Chi‐square = 21.19, *p* = 0.055). Although the *p*value is slightly above 0.05, this may be partly attributed to the relatively lower smoking rates in certain ethnic groups within our sample or the potential underreporting of smoking habits due to social stigma. Additionally, other unmeasured lifestyle or environmental factors may mediate the effect of smoking on CVD risk in this population. Further studies with larger sample sizes and detailed smoking intensity and duration data could provide more insights.

Similarly, the lack of a significant association between physical activity and CVD prevalence in this study contrasts with findings from other regions where physical inactivity is a known risk factor. Previous research in Central Asia has highlighted that physical inactivity is prevalent in Kyrgyzstan, with 21.9% avoidance of physical activity among adults [40] and significant sedentary lifestyle rates among rural populations [12]. This discrepancy might result from the cross‐sectional nature of our data, which limits our ability to assess long‐term activity patterns and their effects on CVD development. Additionally, other contextual factors such as dietary habits, stress levels, or genetic predispositions could be influencing CVD risk independently of physical activity levels. These findings underscore the complexity of CVD risk in Kyrgyzstan, suggesting that further research with longitudinal and larger datasets is needed to clarify the influence of lifestyle factors on CVD in this diverse population.

### 4.6 Ethnicity and CVD Risk Factors

Earlier studies showed evidence for the association between CVD prevalence and ethnicity [7, 11, 12, 47, 48]. Our study examined and presented the relationship between ethnicity and various CVD risk factors, including physical activity, smoking prevalence, fruit and vegetable consumption, alcohol consumption, BMI index (Table 3), gender, age, and education level (Table 4) among different ethnic groups.

Our study showed statistically different CVD prevalence among various ethnic groups, with the highest prevalence among East European origin—42.7%, then Western Asians—39.1%, East Asians—34.2%, Kyrgyzs—30.9%, Central Asians—26.3%, and other minorities—20.6%. These results are consistent with several studies conducted in Kyrgyzstan [11, 12, 44]. The significant subgroup differences observed, particularly between East Asian and other minority‐ ethnic groups, underscore the varied CVD risk landscape within the population. These findings could inform tailored public health strategies for different subpopulations.

The higher share of such particular diseases as HT among Eastern Asian respondents also suggests that ethnicity may play a role in CVD prevalence; however, this association might be driven by different prevalence of risk factors in different ethnic groups. This aligns with regional studies indicating a strong link between HT and lifestyle factors in Central Asia. These findings suggest that interventions targeting blood pressure management may be particularly impactful in reducing overall CVD risk.

Our analysis did not show statistically significant differences in fruit and vegetable consumption, alcohol intake, and BMI among the ethnic groups studied, consistent with the findings of Kontsevaya et al. and Polupanov et al. [12, 44]. However, our analysis showed a statistically significant difference in physical activity among different ethnic groups, with the lowest prevalence in the Kyrgyz group and the highest in the group of other minorities (Table 3). These findings highlight potential disparities in physical activity levels and suggest the need for targeted interventions to promote physical activity to improve cardiovascular health in specific ethnic communities.

According to Pengpid et al., avoidance of physical activity in Kyrgyzstan was 21.9% [40]. Later, Polupanov et al. detected hypodynamia in 15.6% [12] and Kydyralieva et al. in 29.3% [3]. Our analysis showed strong evidence of a statistically significant difference in physical activity among the groups mentioned above (chi‐square = 7.54, *p* = 0.029) with the lowest CVD prevalence in the Kyrgyz group and the highest in the group of other minorities. These findings are essential for identifying potential disparities in physical activity and may be valuable for developing targeted interventions to promote physical activity and improve cardiovascular health in specific ethnic communities.

Our study did not find sufficient evidence to conclude a statistically significant variation in smoking prevalence among the studied groups. However, the borderline *p*‐value suggests a potential trend or link warranting further investigation. Smoking is a long‐known CVD risk factor [3, 21, 42], and even elusive differences in smoking prevalence across ethnic groups may have public health implications. For example, Russian men smoke more compared to men of other ethnic groups in Kyrgyzstan [47].

### 4.7 Gender and CVD Risk Factors Across Ethnic Groups

The associations between gender and CVD risk markers across various ethnic groups are complex. Although there is strong evidence of gender‐related differences in some groups, it did not appear among East Asian, Western Asian, and other minority groups, possibly due to the small sample size. These findings underline the need to consider age‐related differences when analyzing CVD risk factors and the importance of addressing these differences in public health interventions and policies. Further research is needed to explore the underlying mechanisms driving these age‐related associations and develop targeted strategies for promoting cardiovascular health in different age groups and ethnic communities.

### 4.8 Educational Attainment and CVD Risk Factors Across Ethnic Groups

Educational attainment is an important determinant of health outcomes [20, 21, 27, 28, 29]. Poor educational attainment of disadvantaged ethnic minorities predicts poor health literacy, lack of access to relevant information on CVD risk factors, provider–patient communication barriers, poor employment prospects, and economic instability, which in turn act as barriers to healthy living, food security, and healthcare—all strong determinants of CVD [8]. Although educational attainment modifies the relationship between race and CVH, education does not overcome the effect of race [48]. Ethnic differences in CVD risk profiles cannot fully explain the variability in educational attainment [49, 50]. In our study, we did not see any significant association between educational attainment and CVD risk markers within the studied ethnic groups. Further research is needed to understand the potential complexities of the linkage between education level and CVD risk factors within various ethnic contexts in Central Asia.

### 4.9 Strengths and Limitations

Despite the stratified random sampling reflecting the demographic features of the Kyrgyzstani population, the results might have been affected by the relatively small sample size. Although a larger sample size could enhance the statistical robustness of subgroup comparisons, the present sample was deemed adequate for detecting meaningful trends in CVD risk factors by ethnic group in Kyrgyzstan. The power analysis confirmed the sample's capacity to identify significant ethnic group differences within the study's scope. Another limitation is that information on CVD risk factors was mainly self‐reported, introducing response errors and recall bias. This is especially sensitive to socioeconomic level and lifestyle, where participants may not accurately report their income, education level, and daily habits. Self‐reported data could introduce measurement errors and potentially skew the results. For example, individuals may overestimate their physical activity levels or underestimate their intake of unhealthy foods, affecting the assessment of cardiovascular risk factors.

Furthermore, the study might not have considered some crucial variables related to CVD risk, such as access to healthcare, the quality of healthcare, and environmental factors like air pollution or exposure to hazardous substances. The omission of these factors could limit the comprehensiveness of the findings. Additionally, the research may not have adequately accounted for changes over time, as cardiovascular risk factors can vary throughout a person's life. A cross‐sectional study might not capture these variations effectively. Longitudinal studies that track individuals over time can provide more insight into how these risk factors evolve and contribute to the development of CVD.

In conclusion, although stratified random sampling in the study is a valuable approach to reflect the demographic features of the Kyrgyzstani population, it is important to be aware of these limitations in the data collection and analysis. Future research could benefit from larger sample sizes, more objective measures of risk factors, and a broader consideration of variables that may influence CVD outcomes.

### 4.10 Findings of the Study

There is strong evidence of CVD outcomes from lifestyle factors such as BMI, diet, physical activity, alcohol intake, and smoking. It is recognized that CVD prevalence varies across demographic groups, including age, gender, educational attainment, and income. Ethnicity may play a role in CVD prevalence.

Our study highlights that CVD prevalence across diverse ethnic groups can differ significantly. In Kyrgyzstan, the most disadvantaged group in this context is East European, and the least disadvantaged is Western Asian. There are particularities in the effect of lifestyle factors on CVD prevalence in the studied population.

## 5 Conclusion

The current study provides valuable insights into the potential CVD risk factors in the studied population. Although fruit and vegetable consumption, physical activity, and smoking do not show significant differences between respondents with and without CVD, BMI index and alcohol consumption demonstrate statistically significant associations. The distribution of CVD prevalence across various demographic groups suggests that gender, age, ethnicity, and income are associated with CVD prevalence.

There are associations between ethnicity and various CVD risk factors, with significant variations in physical activity among ethnic groups. This highlights the importance of tailoring interventions to address specific needs and overcome barriers faced by different communities. The study reinforces the need for ongoing surveillance and research to better understand the dynamic relationships between ethnicity and CVD risk factors, which could provide valuable insights for public health policymakers, researchers, and clinicians.

## Author Contributions

Conceptualization: K.O., K.D., H.O., and I.V. Methodology: K.O., K.D., and H.O. Software/Data analysis: H.O., H.S.A., M.I., K.O., and K.D. Validation: H.O., H.S.A., I.V., and K.O. Investigation: H.S.A., M.I. Resources: H.S.A. and K.D. Writing (original draft): H.S.A. Writing (review/editing): K.O., K.D., I.V., M.I., and H.O. Visualization: M.I., K.O., I.V. Project supervision: K.O., K.D., and H.O. All authors have read and agreed to the published version of the manuscript.

## Ethics Statement

This study was conducted in accordance with the ethical principles outlined in the Declaration of Helsinki and was approved by the Research Ethics Committee of the International Higher School of Medicine (Approval Number: Ref N10, 28.06.2017). Prior to data collection, written informed consent was obtained from all participants. Participants were fully informed about the purpose of the study, the procedures involved, and any potential risks or benefits associated with their participation. Confidentiality and anonymity of the participants were maintained throughout the study. Personal identifiers were removed or anonymized to ensure that individuals could not be identified in any reports or publications resulting from this research.

## Consent

Informed consent was obtained from all individual participants included in the study. Participants were provided with detailed information about the purpose, procedures, risks, and benefits of the study, and they were given the opportunity to ask questions. Written consent was obtained prior to participation. All data collected were kept confidential and used solely for the purposes of this research.

## Conflicts of Interest

The authors declare no conflicts of interest.

## Permission to Reproduce Material From Other Sources

No material from other sources has been reproduced in this manuscript. All content is original and created by the authors.

## Data Availability

All relevant data are contained within the manuscript and its Supporting Information files.
